# Audiovisual Perception of Lexical Stress: Beat Gestures and Articulatory Cues

**DOI:** 10.1177/00238309241258162

**Published:** 2024-06-14

**Authors:** Ronny Bujok, Antje S. Meyer, Hans Rutger Bosker

**Affiliations:** Max Planck Institute for Psycholinguistics, The Netherlands; International Max Planck Research School for Language Sciences, MPI for Psycholinguistics, Max Planck Society, The Netherlands; Max Planck Institute for Psycholinguistics, The Netherlands; Donders Institute for Brain, Cognition and Behaviour, Radboud University, The Netherlands

**Keywords:** Audiovisual speech, speech perception, lexical stress, prosody, beat gestures

## Abstract

Human communication is inherently multimodal. Auditory speech, but also visual cues can be used to understand another talker. Most studies of audiovisual speech perception have focused on the perception of speech segments (i.e., speech sounds). However, less is known about the influence of visual information on the perception of suprasegmental aspects of speech like lexical stress. In two experiments, we investigated the influence of different visual cues (e.g., facial articulatory cues and beat gestures) on the audiovisual perception of lexical stress. We presented auditory lexical stress continua of disyllabic Dutch stress pairs together with videos of a speaker producing stress on the first or second syllable (e.g., articulating *VOORnaam* or *voorNAAM*). Moreover, we combined and fully crossed the face of the speaker producing lexical stress on either syllable with a gesturing body producing a beat gesture on either the first or second syllable. Results showed that people successfully used visual articulatory cues to stress in muted videos. However, in audiovisual conditions, we were not able to find an effect of visual articulatory cues. In contrast, we found that the temporal alignment of beat gestures with speech robustly influenced participants’ perception of lexical stress. These results highlight the importance of considering suprasegmental aspects of language in multimodal contexts.

## 1 Introduction

Spoken language is most commonly used face-to-face and is thus inherently multimodal. Beside the auditory signal, visual information contributes to speech perception as well (Holler & Levinson, 2019; Perniss, 2018; Rosenblum, 2008). The effect of visual information is well demonstrated by the McGurk effect (McGurk & MacDonald, 1976), where most participants who hear the sound /ba/, while seeing a video of a speaker saying /ga/, perceive the fused percept “da.” Moreover, visual information about articulatory movements improves speech perception in noise (Sumby & Pollack, 1954). This study examines the contribution of two types of visual information to the audiovisual perception of lexical stress, namely, facial articulatory cues and manual beat gestures.

Most of the research investigating visual influences on speech perception has focused on the perception of speech segments, such as vowels and consonants (Massaro, 1987). However, speech consists of more than just segments. Prosody, as cued by suprasegmental information, is also an integral part of human language. Speech rate, lexical tone and lexical stress guide spoken word recognition (Cutler, 2005; Cutler et al., 1997; Maslowski et al., 2019; Mitterer et al., 2011). For instance, lexical stress is contrastive in many languages (e.g., English, Dutch, Spanish) and thus distinguishes segmentally identical words such as Dutch *VOORnaam* (/ˈvoːr.naːm/, [first name]) versus *voorNAAM* (/voːr.ˈnaːm/, [respectable]). Moreover, experiments have demonstrated that lexical stress drives online word recognition and disambiguation (Cutler & Van Donselaar, 2001), even for non-minimal pairs, such as Dutch *OCtopus* and *okTOber* (Pisoni & Remez, 2008; Reinisch et al., 2010). Also, with the inclusion of lexical stress information, words reach the point of uniqueness (i.e., point where no lexical competitors are left) earlier: Without consideration of lexical stress, Dutch words become unique on average after 80% of the phonemes. With lexical stress, the uniqueness point is reached after 66% of the phonemes (van Heuven & Hagman, 1988). Together, these observations highlight the importance of lexical stress in word recognition.

Most studies of the influence of lexical stress on speech perception have only focused on the auditory modality. However, lexical stress is also associated with visual cues. Specifically, the temporal alignment of manual beat gestures has been found to affect audiovisual stress perception. Beat gestures are simple up-and-down gestures of the hand. They are among the most frequently used co-speech gestures (McNeill, 1992). They are usually aligned to acoustically prominent words in utterances (Krahmer & Swerts, 2007; Leonard & Cummins, 2011; Shattuck-Hufnagel & Ren, 2018). This tight temporal coupling seems to be inherent to our speech production, as it can already be found in young children (Florit-Pons et al., 2023). A common assumption is that gestures and speech are planned together in speech production (Kita & Özyürek, 2003). Moreover, the production of upper limb movements affects the acoustic realization of our speech, raising intensity and F0 in the voice (Pouw et al., 2020; Swerts & Krahmer, 2007). This tight relationship between gestures and speech could thus be exploited during speech comprehension.

Work on multimodal spoken language comprehension has mainly focused on representational iconic gestures (e.g., Drijvers et al., 2018), which are distinct from non-representational beat gestures, as iconic gestures (e.g., gesturing “come over here”) convey semantic information (McNeill, 1992). For example, iconic gestures and articulatory cues together support speech intelligibility in adverse listening conditions (Drijvers & Özyürek, 2017). In contrast, beat gestures only convey minimal semantics and are primarily linked to the speech signal in their timing. As such, beat gestures increase the perceived salience of utterances (Krahmer & Swerts, 2007). Furthermore, EEG studies have shown that beat gestures can help focus the listener’s attention on relevant information (Dimitrova et al., 2016) and thus modulate auditory integration (Biau & Soto-Faraco, 2013) and lead to enhanced memory recall (Kushch & Prieto, 2016; Rohrer et al., 2020).

Recently, non-representational beat gestures have also been found to have an effect on low-level speech perception. They can highlight prominent aspects within a word, such as lexical stress. For instance, Bosker and Peeters (2021) presented participants with videos of a talker producing acoustically ambiguous lexical stress tokens of Dutch minimal pairs (e.g., *PLAto* or *plaTEAU*), while producing a large beat gesture aligned to either the first or second syllable. Participants were more likely to perceive lexical stress on the syllable the beat gesture was aligned to. The authors termed this the “manual McGurk effect” (Bosker & Peeters, 2021). This effect was observed in a forced-choice task (i.e., “do you hear *PLAto* or *plaTEAU*?”), in less controlled online testing conditions, in a more implicit shadowing task (i.e., participants more often repeated the ambiguous word as *PLAto* with initial stress when the beat gesture fell on the first syllable than when it fell on the second syllable), and even affected Dutch vowel length perception. Thus, Bosker and Peeters (2021) provided first evidence for how the temporal alignment of beat gestures influences lexical stress perception across a range of tasks.

It is worth pointing out that the talker’s face in the stimuli used by Bosker and Peeters (2021) were masked. This masking was applied to isolate the contribution of gestural timing to stress perception. However, interlocutors in everyday conversation usually also have access to the articulatory movements of their conversational partner, which may be beneficial for processing prosody. For example, a recent study demonstrated how seeing the mouth improves detection of prosodic boundaries (Mitchel et al., 2023). Thus, it remains to be tested how gestural visual information contributes to audiovisual lexical stress perception together with the articulatory cues.

Suprasegmental correlates of lexical stress (e.g., fundamental frequency [F0], intensity, and duration) are less visibly salient on a talker’s face compared with segmental features (e.g., labial place of articulation). Still, producing stress on a given syllable leads to visible changes in articulation. Scarborough et al. (2009) video-recorded native speakers of English producing words that differed in lexical stress (e.g., *SUBject* vs. *subJECT*) while recording their faces with markers on them. They analyzed various measures of facial movement, such as maximum lip opening and chin opening displacement, and found that they were generally larger in stressed syllables. They then presented the videos without any audio to participants in a forced choice task with two alternatives (2AFC task) and observed that the participants could determine the position of primary lexical stress above chance with an average accuracy of 62.2%. This demonstrates that lexical stress is visible on the face to a certain degree in muted videos. Although not tested in Scarborough et al. (2009), these visual cues might in principle influence *audiovisual* perception as well.

Note that in English lexical stress is cued by both suprasegmental and segmental cues, principally vowel reduction. In contrast, in Dutch, segmental changes only play a minimal role in the production of lexical stress (Cutler & Van Donselaar, 2001; Severijnen et al., 2024). Therefore, Jesse and McQueen (2014) tested visual perception of lexical stress in Dutch to determine the visibility of *suprasegmental* cues. They video-recorded a native speaker of Dutch producing Dutch words that were segmentally identical in the first two syllables and only differed in the presence and position of primary lexical stress (e.g., *proJECtor* vs. *projecTIEL*). The first two syllables of the words (e.g., /pro.jεk/) were then presented in video-only format, without any audio, to participants who had to indicate in a 2AFC task which word the speaker meant to produce. The task was different from Scarborough et al. (2009) in that participants were only presented with fragments of polysyllabic words rather than disyllabic lexical stress minimal pairs. Moreover, some of the words had no primary stress in the presented first two syllables (e.g., in *projecTIEL*), arguably making it more difficult to categorize the words. Still, participants’ performance was at approximately 70% accuracy, well above chance.

Taken together, these studies indicate that visual cues to lexical stress are present in articulation and gesture. However, it remains unclear how these cues are weighted by perceivers when combined in more naturalistic audiovisual communication. Specifically, Scarborough et al. (2009) and Jesse and McQueen (2014) only tested the perception of visual articulatory cues to stress under video-only conditions. Whether participants actually use these visible cues when auditory cues are also present is not known. Moreover, both studies used visual stimuli containing only the talker’s face, presenting them at a scale that did not reflect naturalistic conversations. For example, Scarborough et al. (2009) presented the talker’s face at 90% life-size, with participants seated 50 cm away from the screen. As a result, the visual angle (ca. 24.5°) was much greater than encountered in actual face-to-face conversations (ca. 11.5°–3.8°) (Ruijten & Cuijpers, 2020). This could mean that the visual cues in their experiments were more salient compared with the cues in everyday conversations. Alternatively, it is possible that the articulatory cues on the face in fact influence audiovisual perception of lexical stress and help disambiguate ambiguous auditory stress, even if presented at a smaller, more life-like size.

In contrast, beat gestures have been shown to have an effect on *audiovisual* perception (Bosker & Peeters, 2021). In fact, beat gestures can only have an effect in audiovisual contexts, not in video-only stimuli, since their indication of lexical stress is merely the result of their temporal alignment with the auditory signal. However, the gestures used in Bosker and Peeters (2021) were relatively large and quite pronounced, unlike the gestures speakers typically use. It is unclear whether the beat gesture effect persists with smaller, more subtle gestures. Moreover, the face masking in Bosker and Peeters (2021) meant that a potentially important source of visual stress cues, namely articulation, was eliminated. It is unclear what the effect of beat gestures is when gestures are not presented in isolation but with the speaker’s face and articulatory movements.

More specifically, the weighting of articulatory cues to stress and the weighting of beat gestures might interact, potentially enhancing each other’s influence on perception. Drijvers and Özyürek (2017) investigated the influence of visible speech (on the face) and iconic gestures (meaningful representational gestures, produced by the hands; for example, gesturing “driving” by turning an imaginary steering wheel) on degraded speech perception. Participants were presented with videos of a talker producing an action verb in different multimodal conditions (i.e., speech with blurred lips, speech with visible lips, speech with visible lips and manual gesture) for a free-recall task. They found that participants could recall the words more accurately when they had been presented with two visual articulators (i.e., visible lips and gesture) rather than one. Participants thus benefited more from seeing two different visual cues rather than seeing just one. Similarly, we may speculate that seeing beat gesture cues combined with facial articulatory cues improves lexical stress perception, over seeing them in isolation.

Alternatively, presenting a talker with an unmasked face could lead to a reduced effect of beat gestures on audiovisual stress perception. Humans have been found to have a general preference to look at faces rather than other objects in a scene (Ro et al., 2001) and it has been suggested that it is harder to disengage one’s attention from a face than from other stimuli (Mathews et al., 1997; Theeuwes & Van der Stigchel, 2006). Therefore, the presence of a face could lead to less attention being directed at the gestural timing cues and thus reduce their effect on audiovisual stress perception.

It is interesting to note that current models of audiovisual speech perception (e.g., the Fuzzy Logic Model of Perception [see Massaro, 1998; the Supramodal Brain (Rosenblum, 2019)] and multisensory integration in general [Noppeney, 2021]), do not make specific predictions concerning audiovisual prosody. Nor do they speak to the integration of beat gestures, which are only informative in their temporal alignment to the spoken signal. This is because these models were primarily designed and tailored to explain segmental speech perception from “talking faces” (Massaro, 1998), with the classic McGurk effect as its prime example (Magnotti & Beauchamp, 2017). Hence, the present study does not aim to test or discriminate between these models. Instead, it highlights aspects of multimodal speech perception that future models may want to incorporate when accounting for face-to-face spoken communication (see General Discussion).

The present study assessed the influence of articulatory cues on the face and beat gestures in arguably more naturalistic perception of lexical stress than studied in earlier work. Experiment 1 focused only on visual articulatory cues to determine whether they have an effect on *audiovisual* perception of lexical stress. It consisted of an in-house (1A) and an online version (1B) of the same experiment, reported together. Dutch participants were presented with phonetic continua of disyllabic minimal stress pairs (e.g., *VOORnaam* and *voorNAAM*) combined with a video of a talker saying the word with stress on the first or second syllable. Continua were created by interpolating F0 contours, ranging from clear initial-stress (strong-weak; SW) to clear final-stress (weak-strong; WS), while keeping duration and intensity cues ambiguous at average values. In a 2AFC task, participants had to determine the placement of lexical stress by selecting which word of a minimal pair they thought the speaker said (e.g., *VOORnaam* or *voorNAAM*). If visual articulatory cues to stress influence audiovisual perception, participants should be more likely to report hearing stress on the first syllable when the talker in the video produced stress on the first syllable (and vice versa), independent of the phonetic continuum manipulation. Alternatively, they might only use the visual information when the audio is ambiguous with regard to lexical stress. In such a case, we would expect a difference between the two audiovisual conditions only for ambiguous (i.e., mid-continuum) but not for unambiguous audio (i.e., at the continuum end-points). However, if these visual cues are only used in video-only settings (Jesse & McQueen, 2014; Scarborough et al., 2009) but not in audiovisual settings, we should find no difference between the two audiovisual conditions (visual stress on first vs. second syllable), and in fact similar identification curves to audio-only stimuli.

In addition to the audiovisual trials, we included video-only trials to conceptually replicate previous video-only experiments (Jesse & McQueen, 2014; Scarborough et al., 2009) with more realistic presentation size, reflecting face-to-face communication. There we expected participants to perceive the differences in lexical stress from the visual cues on the face alone, as indicated by a higher proportion of SW responses for SW videos (strong-weak, initial stress) and a lower proportion of SW responses for WS videos (weak-strong, final stress).

Experiment 2 combined articulatory cues with beat gestures to determine the relative weights of different visual cues. It consisted of an in-house (2A) and an online version (2B), which are reported together. Participants were presented with the same phonetic continua as used in Experiment 1, together with videos of the talker producing beat gestures on the first or second syllable. Critically, using video-editing, the articulatory cues on the face from Experiment 1 (cueing either SW or WS) were fully crossed with beat gestural alignment (cueing either SW or WS). This allowed us to discriminate the contribution of visual articulatory cues from the contribution of gestural alignment. We expected beat gestures to influence perception similar to Bosker and Peeters (2021), whereby the alignment of the beat gesture to either syllable would shift perception of lexical stress to that syllable. We assumed that this effect would be larger for ambiguous spoken stimuli because the beat gestures would then better disambiguate the auditory signal. For unambiguous speech at continua end-points, we expected a smaller effect since beat gestures would be more redundant and probably weight less heavily.

We decided to run Experiments 1 and 2 in-house and online to determine whether online testing is a viable option for experiments with audiovisual stimuli. With online experiments researchers have less control of various aspects of their study: presentation size for visual stimuli is limited by participants’ screens and audiovisual synchrony can be less reliable and dependent on participants’ internet connection. These factors could possibly affect any visual or audiovisual effects. Therefore, this direct comparison could inform future studies where synchrony and presentation size of audiovisual stimuli are crucial about the potential and limitations of online testing.

## 2 Experiment 1—articulatory cues

### 2.1 Method

#### 2.1.1 Power analysis

We estimated statistical power by means of Monte Carlo simulations (*N* = 1,000; Kumle et al., 2021) using generalized linear mixed models (Bates et al., 2015), setting the overall perceptual difference between videos with lexical stress on the first syllable (“strong-weak” [SW]) and videos with lexical stress on the second syllable (“weak-strong” [WS]) in the audiovisual conditions to 5%, which we considered the smallest, interesting effect size we liked to be able to detect. With this effect size, we achieved a power of 0.81 with 48 participants. See the OSF page (https://osf.io/4d9w5/) for the R code implementing this power analysis.

#### 2.1.2 Participants

Forty-eight native speakers of Dutch were recruited for each version of this experiment (Experiment 1A [in-house]: 37 female, 11 male, median age = 25, range = 19–39; Experiment 1B [online]: 35 female, 13 male, median age = 23, range = 18–37) through the Max Planck Institute for Psycholinguistics participant pool. Participants gave informed consent as approved by the Ethics Committee of the Social Sciences Faculty of Radboud University (project code: ECSW-2019-019). None of the participants reported any hearing or language deficit and all had normal or corrected-to-normal vision. Participants received monetary compensation for participation.

#### 2.1.3 Materials

Materials consisted of seven disyllabic, segmentally identical minimal pairs of frequent Dutch words (see Table 1). The pairs only differed in the position of lexical stress (e.g., *VOORnaam* [first name] vs. *voorNAAM* [respectable]). High-definition video recordings of a male native speaker of Dutch (i.e., the last author) producing all 14 words were made. The speaker was recorded in front of a natural background in a sitting position (see Figure 2). He was instructed to produce the words naturally. Inspection of the acoustic correlates of lexical stress, confirmed the correct stress pattern (see Table 2). Videos were cropped to 620620 pixel squares showing the speaker’s face and torso and exported as avi files. The audio sampling rate was 48 kHz and the video sampling rate was 50 Hz.

**Table 1.** Overview of the Dutch Items Used in This Study [English translations].

SW	WS	IPA transcription
*CAnon* [canon]	*kaNON* [cannon]	/ka.nɔn/
*CONtent* (content [noun])	*conTENT* (content [adjective])	/kɔn.tɛnt/
*SERvisch* [Serbian]	*serVIES* [tableware]	/sɛr.vis/
*VOORnaam* [first name]	*voorNAAM* [respectable]	/vo: r.na:m/
*VOORruit* [windshield]	*voorUIT* [forward]	/vo: r.œyt/
*VOORspel* [prelude]	*voorSPEL* [predict]	/vo: r.spɛl/
*VOORtuin* [front garden]	*forTUIN* [fortune]	/vo: r.tœyn/

Item pairs are segmentally identical (see IPA transcription) and only differ in the placement of lexical stress (indicated by capital letters).

**Table 2.** Mean Acoustic Correlates of Lexical Stress in Our Stimuli.

	Syllable 1		Syllable 2	
	Stressed	Unstressed	Stressed	Unstressed
duration (seconds)	0.37	0.28	0.48	0.41
intensity (dB)	69.66	62.54	65.93	62.36
F0 (Hz)	189.75	114.63	158.94	111.70

**Figure 1.**
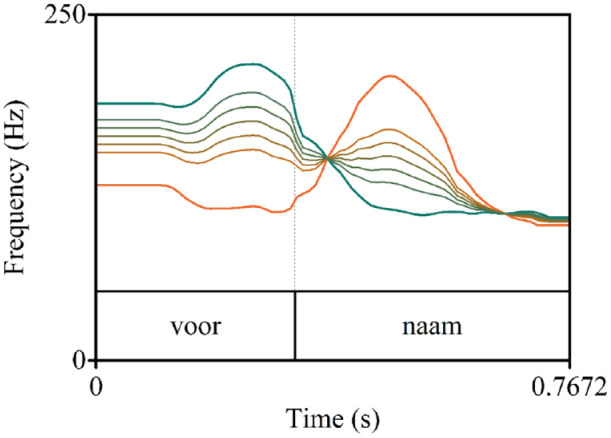
F0 contours of the steps on the stress continuum for the item pair *voornaam.* F0 contours are ranging from unambiguous SW (green) to unambiguous WS (orange), with five more ambiguous steps in between. Steps for each item pair were selected from 11 step continua based on pretesting.

Lexical stress in Dutch is primarily cued by three suprasegmental cues: fundamental frequency (F0), duration, and intensity (Rietveld & van Heuven, 2009). F0 is the biggest contributor in words in isolation and words that align with phrasal accent (Rietveld & van Heuven, 2009). Therefore, we created a lexical stress continuum for each minimal pair (ranging from SW to WS) by manipulating F0, while keeping duration and intensity constant at ambiguous values (i.e., midway between stressed vs. unstressed). The SW and WS audio were extracted from the video recordings and then manipulated. We determined the average duration of the first and second syllable within each item pair and set the values for the syllables in both words to these average values, making intensity and duration identical across words and thus ambiguous with regard to lexical stress. Using duration-ambiguous audio allowed us to linearly interpolate the F0 contours of both words in a pair, creating a single F0 stress continuum per word pair. The F0 contours were sampled at 10 ms time bins and interpolated in eleven steps (Steps 1 and 11 being the original SW and WS contours). Note that this contour interpolation method (see Figure 1) is different from the more artificial F0 interpolation method used in Bosker and Peeters (2021). Specifically, in that study, original F0 contours were removed and replaced with relatively flat F0 contours that only varied in intercept along the continuum steps (i.e., varying only F0 height; not shape). Using the present contour interpolation method resulted in more naturally sounding stimuli.

Interpolated F0 contours were applied to the SW recording (with ambiguous duration and intensity) using PSOLA in Praat (Boersma, 2006). SW and WS recordings were segmentally almost identical, but applying the F0 contour to the SW recordings led to a more natural sounding continuum. This held for all but for one item pair (*SERvisch* vs. *serVIES*), where the F0 contours were applied to the WS recording because it resulted in more natural sounding tokens. These manipulated speech tokens (*N* = 77; 7 pairs × 11 steps) were presented to 10 participants in an audio-only pretest. Participants had to categorize the tokens as either SW or WS in a 2AFC task. Based on their categorization data, we selected five tokens for each pair that sampled a perceptually defined continuum from SW (>80% SW responses) to WS (<20% SW responses) with 3 more ambiguous steps in the middle. In addition, the original recordings (i.e., with unmanipulated F0, duration, and intensity) were used as the extreme ends of the continua, resulting in a total 7-step perceptual lexical stress continuum for each pair.

**Figure 2.**
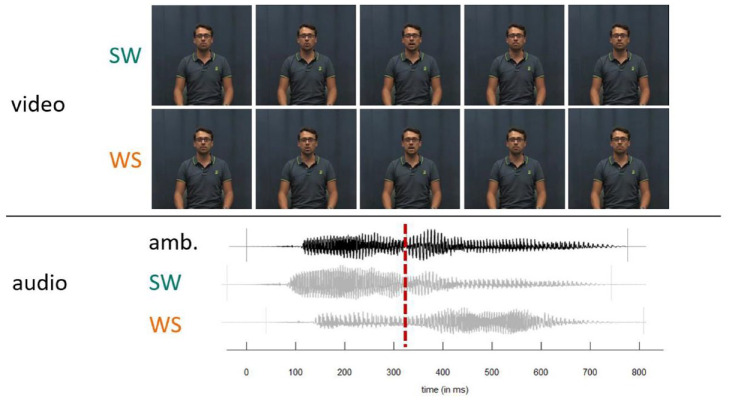
Overview of the AV stimuli. SW and WS videos were combined with every step on the lexical stress continuum. Lower panel shows relative asynchronies of duration manipulated ambiguous audio with original SW and WS audio. Video and audio were aligned at second syllable onset to minimize asynchronies. Note that the visual differences between SW and WS videos are very subtle. SW = strong-weak, stress on first syllable; WS = weak-strong, stress on second syllable.

The experiment had three conditions: audio-only (A), video only (V), and audiovisual (AV). For the A condition, we presented the manipulated F0 continua with a still image of the speaker with a neutral facial expression and a closed mouth. In the V condition, we presented muted videos of the speaker producing either the SW or WS word. Crucially, in the AV condition, we combined each video with the entire lexical stress continuum, aligning the audio and video at the second syllable onset (see Figure 2). This audiovisual alignment was implemented by replacing the original audio in the recorded videos with the manipulated tokens, aligning the manipulated second syllable onset to the timepoint of the unmanipulated second syllable onset. By aligning at second syllable onset, we minimized synchrony issues on either syllable, making the first syllable only slightly misaligned at onset and the second syllable only at offset (see Figure 2). This was the most optimal alignment solution, resulting in only slight audiovisual asynchrony, while at the same time controlling for duration cues (i.e., setting them to ambiguous values midway between stressed vs. unstressed). The average asynchrony for our stimuli was 40 ms at word onset (*SD* = 15 ms, range = 15–77 ms) and 37 ms at word offset (*SD* = 25 ms, range = 6–75 ms) (https://osf.io/4d9w5/), which were deemed acceptable since asynchronies in speech of up to 150 ms are typically perceived as synchronous (Dixon & Spitz, 1980).

All stimuli were cut such that there was approximately a 500 ms silent interval before word onset and after word offset. The average total duration of the stimuli was 1875 ms. Taken together this resulted in 161 items, of which 49 were A items (7 items × 7 steps), 14 V items (7 items × 2 videos), and 98 AV items (7 items × 7 steps × 2 videos), each presented once for reasons of time.

#### 2.1.4 Design and procedure

The in-house experiment, Experiment 1A, was run in Presentation® software (Version 18.0, Neurobehavioral Systems, Inc., Berkeley, CA) and presented on a 24′′ full HD screen with a refresh rate of 144 Hz. AV stimuli appeared in the center of the screen as 1080 × 1080 pixel displays on a white background. Audio was presented through high quality headphones (beyerdynamic DT 770 PRO 32 Ohm) at a comfortable volume. Participants were seated at a distance of approximately 60 cm from the screen. The videos were presented at full screen making the speaker’s head 5.7 cm wide and 7.5 cm tall. From the distance to the screen (*d*) and the size of the head (*h*) we could calculate a visual angle (*θ*) indicating how big the head appeared to the participants, *tan(θ/2)* *=* *(h/2)/d*. The visual angle of the head was 7.15°, which is equivalent to a conversation with someone at 1.93 m distance, assuming an average male head height of 24.1 cm from the chin to the top of the head (Lee et al., 2006). This falls in the range of interpersonal interactions (Ruijten & Cuijpers, 2020) and is considered a comfortable interaction distance.

For the online version of this experiment, Experiment 1B, we used Gorilla Experiment Builder (http://gorilla.sc; Anwyl-Irvine et al., 2020). In Experiment 1B, presentation size was determined by the participants’ screen. Presentation size was maximally equal to Experiment 1A if the screen was large enough. Otherwise, presentation size was restricted to the height of the screen. Devices such as tablets and phones were not allowed for the experiment. In a brief questionnaire preceding the experiment, participants reported an average seating distance of 50.9 cm and an average screen height of 17.72 cm. This resulted in an average visual angle of 5.18°, which is equivalent to a conversation with someone at a distance of ~2.66 m, but there was considerable variation in visual angle between participants. See OSF for participants’ reported screen sizes and calculated visual angles (https://osf.io/4d9w5/). Finally, participants were required to use high quality headphones, which was checked with a headphone screening prior to the main experiment (based on Huggins Pitch, see Milne et al., 2021).

In both Experiment 1A and 1B, all 161 unique items, from the three conditions (14 AV, 7 A and 2 V for each item), were presented once in a fully randomized order. This meant that A, V, and AV stimuli were intermixed. The task was to decide from two words presented on screen, what the speaker was saying (2AFC). Before the task, participants received four practice trials to become familiar with the materials and the task. Four stimuli sampled from all three conditions were chosen as practice trials, using only original unmanipulated audio (A-SW, V-WS, AV-SW AV-WS). Participants were instructed to look at the screen at all times. They were explicitly told beforehand that they would see videos with and without audio, and audio with a still image.

A trial began with the two response options (e.g., *VOORnaam* vs. *voorNAAM*) presented on either side of the screen (Arial, font size 16) for 1,500 ms. Lexical stress was indicated by capital letters. The sides on which SW and WS words were presented were counterbalanced across participants. Then, a fixation cross was displayed for 500 ms, and then the stimulus. The fixation cross was positioned at the center of the speaker’s mouth, which appeared 120 pixels above the center of the screen. After the stimulus, the response options appeared again for a maximum of 4,000 ms. Participants responded by pressing the “Z” and “M” button on the keyboard, corresponding to the left and right word on screen. After a response, the selected word was highlighted by displaying it in a bigger font size (20) for 500 ms. After this, a 500-ms blank screen was presented before the next trial began automatically. Halfway through, participants had a chance to take a break. The duration of the experiment was approximately 40 min.

Note that all A, V, and AV trials were presented in fully random order. We decided to present all trials intermixed to ensure participants would look at the screen throughout an experimental session (i.e., not closing their eyes), since V trials could only be categorized by watching the screen. However, this is different from earlier work (Jesse et al., 2017; Scarborough et al., 2009) that only tested V-only stimuli. Therefore, to assess the effect of this intermixed order on participants’ responses, we additionally included a V-only task after the main experiment. Hence, participants were presented with the V-only stimuli in the main block of the experiment, where they were intermixed with the A and AV trials, and again in a separate V-only block. After completing the main block, participants of Experiment 1A were presented with three rounds of all the V items in a randomized order. Due to a scripting error, the V items were presented only once in Experiment 1B. The purpose of the V-only task was to more closely replicate the experiment by Jesse and McQueen (2014), who only used V-only stimuli, and determine the effect of the articulatory cues when participants could solely focus on the visual information and would not have to switch between modalities.

### 2.2 Results

Results for Experiment 1A and 1B were analyzed together with generalized linear mixed models using the lme4 library (Bates et al., 2015) in *R* (R Core Team, 2021). Two different models were created, one for the V condition and one comparing the AV condition to the A condition. In both models, participants’ categorization responses, that is lexical stress on the first (SW coded as 1, for example, *VOORnaam*) or second syllable (WS coded as 0, e.g., *voorNAAM*), formed the dependent variable. Models with Version (i.e., in-house vs. online; see OSF: https://osf.io/4d9w5/) as a predictor neither revealed significant effects of Version nor increased log likelihood model fit, indicating similar response patterns for the in-house and online versions. Hence, we report the simpler models without Version as a predictor.

**Figure 3.**
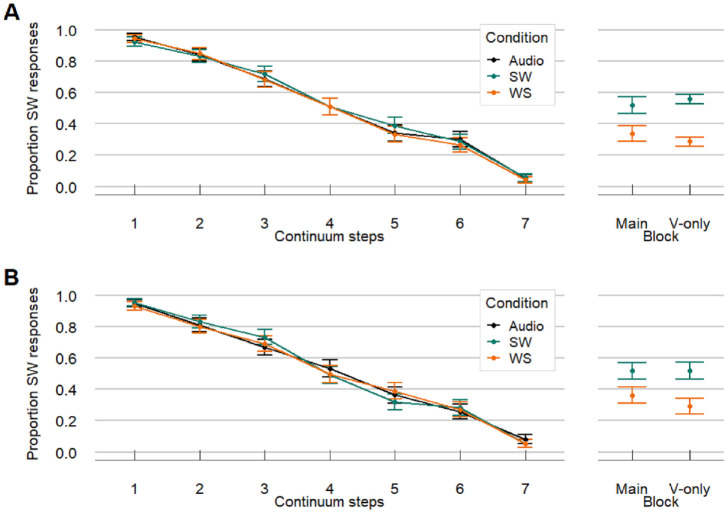
Results from Experiment 1: Comparison of in-house results (1A) and online results (1B) reveal similar patterns (note: combined data were analyzed in the statistical models). Left panels show the results from the AV and A-only trials. Proportion SW responses were highest for auditory Step 1 (unambiguous SW) and lowest for auditory Step 7 (unambiguous WS). The colored AV lines overlap with the black A-only line, suggesting that visual articulatory information on the face have little influence on audiovisual perception of lexical stress. However, the right panel shows that participants could in fact see differences in lexical stress when presented with the videos only, without any audio. Moreover, the effect in the unimodal V-only block was bigger than in the main block. Note that the effect on V-only trials appears to be driven mostly by the WS pattern. SW = strong-weak, stress on first syllable; WS = weak-strong, stress on second syllable.

We ran the video-only model to assess whether participants could reliably use the visual cues to lexical stress in video-only trials, aiming to replicate findings by Jesse and McQueen (2014). This video-only model included Video (categorical, deviance coded: SW as 0.5 and WS as −0.5), Block (categorical, deviance coded: V-only block as 0.5 and main block as −0.5) and their interaction as predictors. Participant and Item were included as random effects including random slopes for Video and Block. The video-only model revealed a significant effect of Video (β = 0.977, *SE* = 0.189, *z* = 5.173, *p* < .001), meaning that there was a significant difference between the proportion SW responses for SW and WS videos. The intercept also turned out significant (β = −0.356, *SE* = 0.133, *z* = −2.671, *p* = .008) indicating that participants’ responses were generally biased toward WS (see right panel Figure 3). This means that differences in lexical stress were indeed visible in the videos, although largely driven by the WS videos. Moreover, there was a significant interaction of Video and Block (β = 0.393, *SE* = 0.148, *z* = 2.656, *p* = .008) showing that the Video effect was larger in the unimodal video-only block than in the main block (see right panel Figure 3). A model including Version (categorical, deviance coded, online as 0.5 and in-house as −0.5) was rejected as it did not improve the fit, indicated by log-likelihood ratio tests. Hence, the results for the video-only trials were comparable between the in-house and online version.

Next, we compared the audiovisual conditions to the audio-only condition. In this model, we included Continuum Step (continuous; z-scored by taking steps 1-7, subtracting the mean, and dividing by the Standard Deviation) and Condition (categorical predictor with three levels; SW, WS, and A; with A mapped on the intercept), with Participant and Item as random effects with random slopes for both predictors. The model only showed a significant effect of Continuum Step (β = −1.865, *SE* *=* 0.125, *z* *=* −14.908, *p* < .001) meaning that with increasing steps on the continuum the proportion of SW responses decreased. However, neither SW videos (β = 0.004, *SE* *=* 0.064, *z* *=* 0.058, *p* = .954) nor WS videos (β = −0.054, *SE* *=* 0.068, *z* *=* −0.810, *p* = .418) influenced the responses when compared with the A condition (intercept). The responses on AV trials were hence similar to A trials, which is illustrated by the overlapping lines in the left panel in Figure 3.

Similarly, to the video-only model, the addition of Version (in-house vs. online) did not improve fit for the audiovisual model, suggesting similar performance in-house and online. Moreover, adding an interaction between Continuum Step and Condition to the model did not significantly improve the model fit to the data, as revealed by log-likelihood model comparison.

### 2.3 Interim discussion

Results from the V-only block replicated the findings by Scarborough et al. (2009) and by Jesse and McQueen (2014). The results showed that participants could differentiate the position of lexical stress from seeing muted videos alone. In our data, this effect appeared to be driven by visual cues in the WS condition. Responses for SW videos were around chance level in all blocks, except for the V-only block in Experiment 1A. This indicates that the visual cues for lexical stress were generally more apparent in WS words. Since a WS stress pattern is less frequent in Dutch disyllabic words (van Heuven & Hagman, 1988), it is possible that the speaker produced the visual stress cues in the WS words more clearly than in the more common SW words. Alternatively, more atypical stress patterns might be perceptually more salient to the participants and help categorization.

Moreover, we found that the video effect was smaller in the main block, when V trials were intermixed with A and AV trials. The smaller effect likely reflects the increased difficulty due to modality switching. Especially switching from auditory processing to visual processing has been found to be very costly (Sandhu & Dyson, 2012). Alternatively, the difference in effect size could be due to the presentation order since the V-only block was always presented after the Main block. Hence, participants were more familiar with the items in the V-only block, where we found a larger effect.

Regardless, we found a video effect in both blocks and both experiments, meaning that participants could perceive a difference between silent videos with different lexical stress patterns. Moreover, they could do so even at smaller and arguably more realistic presentation sizes for face-to-face communication than in previous studies (Jesse & McQueen, 2014; Scarborough et al., 2009). In Experiment 1A, we presented the videos at a visual angle equal to a realistic conversation distance of 1.9 m. In Experiment 1B, the presentation size was more variable and generally smaller, simulating a conversation with someone at a distance of 2.66 m. While not statistically significant, our data show that the effect was numerically stronger in Experiment 1A with the larger presentation. The reasons for this unexpected difference are unknown. It is possible that the perceptibility and usability of some visual stress cues are affected by the visual angle, and that some cues are affected more strongly by presentation size than other. For example, SW stress cues might be more sensitive to presentation size than WS stress cues. Still, we found the Video effect in both versions, indicating that subtle lexical stress cues on the face can affect visual perception at varying scales.

However, despite these informative visual cues in the videos, we failed to find a video effect in the AV condition. We did not find evidence for visual information on the face affecting perception of lexical stress. This null result cannot be fully explained by any visual properties of the articulatory cues in the videos themselves, such as low salience, since we found that participants were able to perceive visual cues in the muted videos. Hence, we would expect to find a video effect in audiovisual perception, at least for audiovisual trials with WS video (showing the largest V-only effect). However, we did not find such an effect. This result will be further considered in the General Discussion.

## 3 Experiment 2—articulatory cues and beat gestures

In Experiment 2, we investigated the effect of the temporal alignment of manual beat gestures on the audiovisual perception of lexical stress when presented with facial articulatory cues at the same time. Even if articulatory cues have little effect on audiovisual stress perception, the presence of a visible face could detract attention from the beat gesture and thus reduce the effect of beat gestures compared with earlier studies.

### 3.1 Method

#### 3.1.1 Participants

All participants were native speakers of Dutch and recruited from the Max Planck Institute for Psycholinguistics participant pool (Experiment 2A [in-house]: *n* = 48; 36 female, 10 male, 2 other; median age = 22; range = 18–30; experiment 2B [online]: *n* = 51; 38 female, 13 male, median age 25, age-range = 18–38). Participants from the previous experiments were excluded from participation. Participants gave informed consent as approved by the Ethics Committee of the Social Sciences department of Radboud University (project code: ECSW-2019-019). None of the participants reported any hearing or language deficit and all had normal or corrected-to-normal vision. Participants received compensation for participation.

#### 3.1.2 Materials

**Figure 4.**
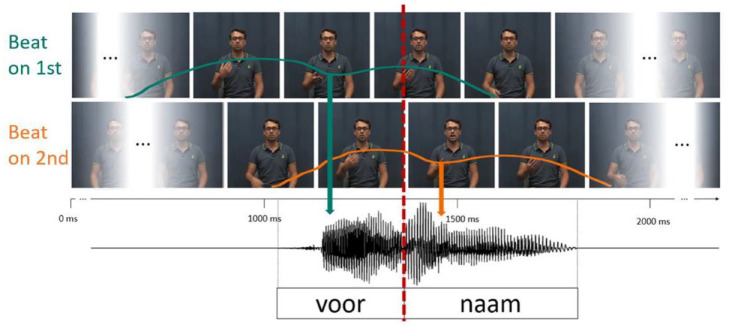
Head from the non-gesture videos from Experiment 1 (left image) was pasted onto a video of the speaker producing a beat gesture (middle image) resulting in a seamlessly combined stimulus (right picture).

The videos from the first experiment, containing visual articulatory cues to lexical stress, were combined with new recordings of the speaker, producing beat gestures on either the first or second syllable, to test the effect of articulatory cues and beat gestures on audiovisual lexical stress perception. The videos with beat gestures were recorded in the same session as the videos previously used in Experiment 1. The speaker was asked to produce all items again, while also producing a beat gesture, which naturally aligned with the word’s stressed syllable. Specifically, from a resting position with the palm facing down, the speaker rotated his hand forward and upward, indicating the apex of the gesture with the palm facing up, before returning back to a resting position (see Figure 4). In comparison to Bosker and Peeters (2021), the beat gestures in our experiment were relatively small and subtle (see Figure 4, https://osf.io/4d9w5/). For instance, our talker produced gestures with a smaller peak deceleration (i.e., less abrupt “stop” of the gesture). We quantified the gestures’ peak deceleration by the vertical height (in percent of the video height) of the index finger from the highest to the lowest point of the gesture. In our stimuli, the gestures traveled 17% in 180 ms (9 frames, 43% to 26%), where in Bosker and Peeters (2021) it was 36% of the video height in 180 ms (9 frames; from 65% to 29%).

**Figure 5.**
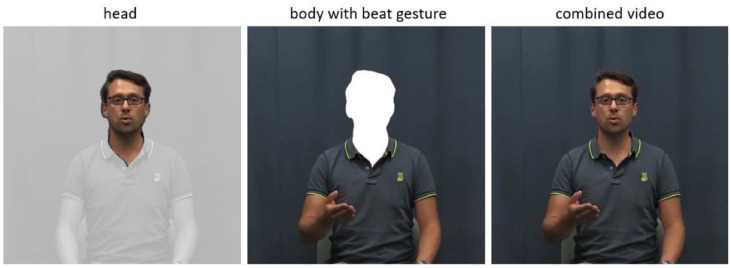
Overview of the stimuli in Experiment 2. Naturally produced beat gestures with apex aligned to the first (SW = strong-weak, green) or second syllable (WS = weak-strong, orange). Colored lines illustrate the hand trajectory in SW and WS video, with approximate alignment with the speech signal. Videos were combined with all steps of the auditory stress continuum. Video and audio were aligned at second syllable onset.

We then created manipulated video stimuli using Adobe Premiere Pro CC 2018. We cut out the head (including the neck) from the videos without a beat gesture (from Experiment 1) and pasted it onto a video of the speaker producing either word of the same item (e.g., *VOORnaam* or *voorNAAM*) with a beat gesture. Since both videos were recorded back to back, the videos aligned very well. In some cases, the two videos were slightly misaligned due to minimal talker movement. Such misalignments were adjusted frame by frame by minimally moving the head horizontally to align with the body on all frames. Finally, a feathered mask obscured any hard edges. This resulted in a seamlessly merged video (see Figure 5).

The apex of the beat gesture was temporally aligned to the vowel onset of either the first or second syllable by moving the video signal presenting the beat gestures (i.e., the torso) forward/backward in time (see Figure 4). This manipulation allowed us to fully cross facial cues producing SW or WS items with a beat gesture cuing either SW or WS, thus also creating incongruent combinations (e.g., face saying SW but gesture cuing WS). All stimuli were cut such that there was approximately a 750 ms silent interval before word onset and after word offset. This ensured that the beat gesture was not cut off and could be seen from beginning to end. The average duration of the stimuli was 2,375 ms. These combined videos were then again combined with the entire lexical stress continuum, resulting in a total of 196 audiovisual stimuli (2 heads × 2 beats × 7 steps × 7 items).

#### 3.1.3 Design and procedure

The design and procedure were identical to Experiment 1, aside from the stimuli presented. In Experiment 2, only audiovisual (AV) stimuli were presented. All 196 audiovisual stimuli, plus an additional 28 catch trials, were presented to the participants. The catch trials were meant to keep participants’ attention on the screen. Specifically, in contrast to Experiment 1, participants in Experiment 2 could in principle perform the experiment with their eyes closed (against instructions). To motivate multimodal perception, catch trials were interspersed with the experimental AV trials, which forced participants to keep watching the screen attentively. Catch trials involved 28 visually congruent stimuli (e.g., face and beat indicating unambiguous SW or unambiguous WS) with congruent unambiguous auditory stress (Step 1 or 7), but with a big white fixation cross drawn on the speaker’s face. On such trials, participants were instructed *not* to identify which word they heard but *instead* to press the spacebar whenever they saw a white cross on the speaker’s face. Hence, accurate performance (i.e., pressing spacebar) on catch trials could be taken as an index of continuous watching of the screen. Before the task, participants received six practice trials including 2 catch trials to become familiar with the materials and the task.

The in-house and online experiments were identical in design and procedure. The screen specifications of the in-house Experiment 2A were identical to those of Experiment 1A. In the online Experiment 2B, the presentation size depended on the size of the screen participants were using. The average screen size was ca. 1502 × 743 pixels, making the presentation size of the videos only approximately 69% of the size of the in-house experiment. Participants were only allowed to participate in the experiment with high-quality headphones, which was checked with a headphone screening prior to the main experiment (based on Huggins Pitch, see Milne et al., 2021). The duration of the experiment was approximately 40 min.

### 3.2 Results

Data from Experiment 2A and 2B were analyzed together to compare outcomes from the in-lab and online versions. Regarding catch trial performance, all participants self-reported having looked at the screen throughout the experimental session. The catch trial accuracy was high (mean accuracy = 0.90; *SD* = 0.16) but with some interindividual variation (see https://osf.io/4d9w5/ for raw accuracy data).

**Figure 6.**
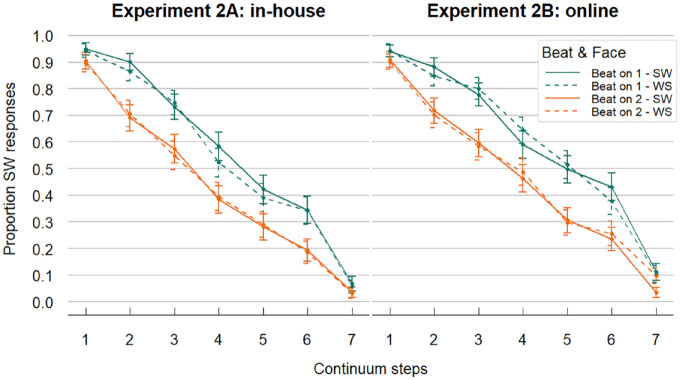
Experiment 2 data: Left and right panels show results from the in-house and online experiment respectively (note: combined data were analyzed in the statistical models). Online responses were slightly more biased toward SW responses, but otherwise the results were very similar. Proportion SW responses decreased when auditory steps sounded more WS-like. When participants saw a beat gesture aligned to the first syllable (Beat on 1, green lines), the proportion SW responses was higher across all steps (and vice versa). Overlapping dashed (Face = WS) and solid lines (Face = SW) suggest that articulatory cues on the face had little influence on audiovisual lexical stress perception. SW = strong-weak, stress on first syllable; WS = weak-strong, stress on second syllable.

Overall, response patterns were similar in the in-house and online versions (see Figure 6). The proportions of SW responses went down with increasing steps on the phonetic continua (audio becoming more WS-like). Moreover, beat gestures appear to shift responses in the direction of the stress pattern they indicated; that is, beat gestures aligned to the first syllable (SW-biasing) led to a higher proportion of SW responses and beat gestures aligned to the second syllable (WS-biasing) led to a lower proportion of SW responses (difference between orange and green lines). Dashed and solid lines, on the other hand, overlap, suggesting little to no effect of facial articulatory cues.

Data for inferential statistics were analyzed with generalized linear mixed models. A model with participants’ categorization responses, that is lexical stress perceived on the first (SW coded as 1, for example, *VOORnaam*) or second syllable (WS coded as 0, for example, *voorNAAM*) as the dependent variable, was created. Continuum Step (continuous; z-scored), Beat (categorical predictor, deviance coded: SW as 0.5 and WS as −0.5), Face (categorical predictor, deviance coded: SW as 0.5 and WS as −0.5), and Version (deviance coded: in-house as 0.5 and online as −0.5) were included as predictors, together with interactions of Beat and Face, and Version with all other predictors. The model also included random intercepts for Participants and Items and by-participant and by-item random slopes for all predictors.

The model revealed a significant effect of Continuum Step (β = −1.741, *SE* = 0.135, *z* = −12.915, *p* < .001) showing decreasing proportions of SW responses with increasing continuum steps (sounding more WS-like). The predictor Beat also turned out significant (β = 0.854, *SE* = 0.13, *z* = 6.575, *p* < .001), indicating higher proportions of SW responses whenever the beat gesture was aligned to the first syllable. On the other hand, Face was not significant (β = 0.001, *SE* = 0.057, *z* = 0.148, *p* = .882). Finally, only a main effect of Version was significant (β = 0.25, *SE* = 0.122, *z* = 2.051, *p* = .04), meaning that the intercept was somewhat higher in the in-house version compared with the online condition. However, no interaction effects were found, suggesting similar performance in in-house versus online testing, mirroring Experiment 1.

To test whether the effect of either visual channel (beat gestures and/or facial articulation) was larger for more ambiguous continuum steps (i.e., in the middle of the categorization curves), we created a more complex model with QuadraticStep (i.e., z-scored Step squared, continuous) and its interactions with Beat and Face as predictors. This model marginally improved the model fit as indicated by log likelihood comparison, χ^2^(8) = 15.444, *p* = .051. In that model, QuadraticStep interacted significantly with Beat (β = −0.134, *SE* = 0.05, *z* = −2.707, *p* = .007). This suggests a slight effect of beat gestures influencing the perception of lexical stress more, the more ambiguous the auditory cues are.

### 3.3 Interim discussion

Results showed a clear effect of auditory information on the perception of lexical stress. The proportions of SW responses were highest for the low end of the phonetic continua (i.e., Step 1) and decreased along the continua as the steps sounded more WS-like. In addition, we replicated previous findings of beat gestures influencing lexical stress perception that had been obtained with the talker’s face masked (Bosker & Peeters, 2021). Our participants responded with higher proportions of SW responses when the beat gesture indicated an SW stress pattern (i.e., aligned to the first syllable), and lower proportions when beat gestures indicated a WS pattern (aligned to the second syllable). Finally, we found some indication that beat gestures do influence lexical stress perception differently, depending on the ambiguity of the audio. That is, beat gestures seemed to have their strongest impact when the audio was in the ambiguous range, approximately between 20% and 80% of SW responses. Still, even though their effect was slightly reduced at the continua extremes, beat gestures influenced perception across all steps (visible as separation of green versus orange lines at Steps 1 and 7 in Figure 6), indicating they are a rather ubiquitous cue of lexical stress. This is in stark contrast to articulatory cues to stress that—mirroring Experiment 1—did not show reliable effects in audiovisual stress perception.

## 4 General discussion

This study aimed to determine whether perceptible visual cues to lexical stress on the face influence *audiovisual* stress perception (Experiment 1). In addition, in Experiment 2, we tested the combined effect of visual cues on the face and beat gestures on audiovisual stress perception. That is, we tested whether the temporal alignment of beat gestures to spoken syllables affected audiovisual stress perception in more naturalistic situations (i.e., without face masking).

Our video-only results showed that participants could distinguish minimal stress pairs from just seeing a speaker’s face. This study thus replicates previous findings (Jesse & McQueen, 2014; Scarborough et al., 2009). Although Jesse and McQueen (2014) used a slightly different task in which participants would sometimes be presented with word fragments without primary lexical stress, their results support the same conclusion, namely that participants can perceive and use visual stress cues on the face. In addition, our experiment showed that this effect persisted even when the face was presented at a smaller scale, mimicking face-to-face interactions. Moreover, we found this effect when V trials were intermixed with A and AV trials, requiring task switching, and we found it both in in-house and online settings. However, our effect size in V-only stress perception was relatively modest compared with Jesse and McQueen (2014), which may be attributed to these design changes.

All in all, our results highlight that there were visible cues to stress on the face in our stimuli, primarily when an WS stress pattern was produced, that the participants could perceive. However, none of the experiments found evidence that facial articulatory cues of lexical stress influenced *audiovisual* stress perception. This held when specifically testing facial articulatory cues (Experiments 1A & 1B) and when we combined facial with gestural cues (Experiment 2A & 2B). The lack of evidence cannot be accounted for by mere absence of visual cues to stress, since we observed unimodal effects of video in the V-only condition in Experiments 1A and 1B. That is, participants could distinguish between videos of the talker producing either an SW or WS word, indicating that there were visual cues supporting this categorization.

We suggest two explanations for the lack of an effect of articulatory cues on *audiovisual* stress perception. One explanation is that the additional processing of auditory information causes an automatic downregulation of attention to the subtle visual cues. That is, participants perceive the visual information less accurately in an audiovisual context. This is in line with findings that audiovisual integration of speech falters when participants allocate their attention to an auditory task (Alsius et al., 2005). Alternatively, people might still perceive the visual information as accurately as in the V-only condition but weight it less heavily in audiovisual integration (Mozolic et al., 2008).

Indeed, people can actively weight visual and auditory cues in audiovisual perception, depending on the communicative context. For example, if access to the auditory signal is hindered (e.g., in loud background noise), participants tend to show a larger McGurk effect (Stacey et al., 2020). Therefore, future research may try to disentangle these two accounts by adding background noise to the target speech. In our experiment with clear speech, salience could have biased participants toward auditory-dominant processing. But according to the multisensory cue-weighting account, participants may actually upweight the visual cues in audiovisual stress perception as the auditory cues become less accessible (i.e., masked). Such cue-weighting of multimodal prosody has previously been demonstrated for phrasal intonation differentiating questions versus statements (Miranda et al., 2021), and could also play a role in lexical stress perception in noise (Mok, 2022). Therefore, we do not claim that visual cues to prosody never influence audiovisual speech perception, but we couldn’t find any evidence for it when testing speech-in-quiet in our study.

Next to flexible multisensory cue-weighting, it may be that some participants rely on these visual prosody cues more than others (see https://osf.io/4d9w5/ for individual by-participant variation in Experiment 1). Further research could investigate individual differences in multisensory cue-weighting (Wilbiks et al., 2022). Also, languages vary in how stress is cued acoustically. Specifically, while Dutch stress involves primarily suprasegmental cues (e.g., F0, intensity, and duration) (Severijnen et al., 2024), in English vowel reduction is a strong cue to unstressed syllables (Cutler, 2015). This segmental reduction in the English stressed versus unstressed contrast may be visually more salient compared with only suprasegmental cues in Dutch (Scarborough et al., 2009). Hence, future experiments could test whether visual cues to stress in English (i.e., visual correlates of segmental *and* suprasegmental stress cues) do influence audiovisual stress perception. Yet, in our data on Dutch lexical stress, we did not find any reliable evidence demonstrating that visual articulatory cues influenced audiovisual stress perception.

In contrast, we found clear evidence that beat gestures do influence lexical stress perception. This supports previous findings of this “manual McGurk effect” (Bosker & Peeters, 2021). Importantly, there were a few key differences between Bosker and Peeters (2021) and our study. Bosker and Peeters (2021) presented participants with videos of a talker who produced larger and more pronounced beat gestures. Also, the critical target words were embedded in a lead-in sentence (“Now I say the word. . .”) that itself also contained beat gestures. In our study, the gestures were smoother, with less emphasis on the apex, presented on isolated words. Nevertheless, we observed a comparable effect size across the two studies (overall shift in proportion SW responses of about 0.2). The fact that we still found an effect makes a strong case for the importance of beat gestures in audiovisual speech perception, even when the gestures are less pronounced. Furthermore, it indicates that the form and salience of the gesture are less important than the temporal alignment of the gesture with the spoken signal. Future work could focus on even more natural gestures, for example spontaneous gestures produced by a naïve talker.

Another major difference to Bosker and Peeters (2021) was the fact that we presented videos with the talker’s face unmasked. Despite the face clearly providing visual (and sometimes conflicting) information with regard to lexical stress, this did not interact with the gestural effect. We hypothesized that two different visual cues (e.g., articulatory cues and beat gestures) could enhance audiovisual perception beyond the influence of just one visual cue, similar to previous findings on segmental speech and iconic gestures (Drijvers & Özyürek, 2017). However, facial cues to suprasegmental contrasts are much less salient, which could in part explain why we were not able to find an effect of articulatory cues in Experiment 1. By extension, it was unlikely that these articulatory cues would enhance the gestural effect.

In contrast, it was also possible that the presence of an unmasked face, especially in combination with a smaller and less salient beat gesture, would attenuate the effect of beat gestures on stress perception. Faces tend to be looked at more than other objects in a scene (Ro et al., 2001) and they draw more attention (Mathews et al., 1997; Theeuwes & Van der Stigchel, 2006). Presentation of the face could thus have reduced attention to the beat gesture affecting the results. However, we find a similar beat gesture effect as Bosker and Peeters (2021), suggesting that it is a robust effect and likely not largely affected by face (un)masking. However, future studies could test this suggestion more explicitly by presenting the same video stimuli with and without face masking.

Results from our online experiments (Experiments 1B and 2B) were very similar to our in-house experiments (Experiments 1A and 2A). Importantly, the beat gesture effect was not smaller in the online experiment even though the presentation size was smaller due to the limitations of online testing. This is strong evidence for the influence of beat gestures, as the effect is equally large when presented at life-like size, imitating a face-to-face conversation at a distance of 1.93 m, as well as when presented significantly smaller. This suggests that the effect of beat gestures is ubiquitous and very robust. Moreover, audiovisual synchrony is presumably more variable in online experiments than in fully controlled in-house experiments. Our study’s results suggest that the gesture effect is robust across variable audiovisual asynchronies. However, this remains to be tested in future work manipulating gesture-speech synchrony in a more fine-grained manner. Nonetheless, we can conclude that online testing is a viable option for studies using audiovisual stimuli, where presentation size and synchrony might be crucial.

We aimed to investigate audiovisual perception of lexical stress in a more naturalistic setting compared with earlier work, which we achieved mostly through our stimuli and their presentation size. All words and gestures were produced naturally and videos were presented at realistic sizes, which mimic daily face-to-face interactions, with the face unmasked. The interpolation of the F0 contours further increased the naturalness of the stress continuum when compared with Bosker and Peeters (2021), who used artificial F0 contours. However, it was still an experimental lab-based study with its own limitations. While the recordings were produced naturally, the talker was asked to sit still, which might have minimized production of non-articulatory visual cues such as head nods and eye-brow movements, which have been found to be strong cues of prominence (Swerts & Krahmer, 2008). Moreover, we only presented single words. It remains to be seen whether the effect of beat gesture remains in a disambiguating sentence or discourse context. If semantic or syntactic cues constrain word recognition and thus disambiguate a word, less weight might be assigned to the beat gesture and thus the effect could appear smaller in such richer contexts. Moreover, in a sentence context, lexical stress might interact with sentence-level prosody. Crucially, the word might not always appear in focused positions but out-of-focus instead (e.g., discourse-old, already mentioned referents). For words in such non-focused positions, beat gesture integration has been found to be more costly (Dimitrova et al., 2016). Therefore, it is possible that the effect of beat gestures may differ depending on the position of a word within a sentence.

While we did not set out to test any specific models of audiovisual speech perception, our findings do have implications for such models. Specifically, some models of audiovisual speech perception, for example the Fuzzy Logic Model of Perception (FLMP; see Massaro, 1998), assume that visual and auditory information are identified separately and matched to learned prototypes in parallel (e.g., how much a phoneme matches an abstract prototype representation) before they are integrated into one multisensory percept. However, beat gestures have no inherent meaning. Not the beat gesture itself, but rather its temporal alignment to speech informs a listener on the position of lexical stress. This makes beat gestures unique visual articulators. It is unclear how a model like the FLMP that assumes unimodal processing first would deal with beat gestures.

The Supramodal Brain (Rosenblum et al., 2017) is a model that assumes that the multisensory speech cues that we perceive are not tied to a specific modality and are thus processed supramodally. It would suggest that a beat gesture is not processed independently as a visual cue alone but rather together with the auditory (and other multimodal) cues. The main difference with the FLMP is the timing of the integration of information from different modalities. The FLMP proposes a rather late integration, whereas the Supramodal Brain assumes early integration. Examining the time-course of the Manual McGurk effect (e.g., with eye-tracking) could give us unique insights into audiovisual integration of speech. Are beat gestures used to guide online word recognition (i.e., beat gesture information is used as soon as it is available) just like acoustic cues to prominence (Reinisch et al., 2010), or are beat gestures only used post hoc after the auditory speech has been processed independent of the beat gesture? Answers to these questions would inform models of audiovisual speech perception on the timing of gesture-speech integration and give us a better understanding of the role of gestural timing in speech perception.

In conclusion, lexical stress perception is not a unimodal auditory process but is inherently multimodal. We found that beat gestures had a large and robust effect on lexical stress perception, suggesting they can play a role in audiovisual communication. Therefore, researchers should consider the rich multimodal context of human communication when studying the perception of lexical stress and other segmental and suprasegmental aspects of speech.
